# Lessons learned from over a decade of data audits in international observational HIV cohorts in Latin America and East Africa

**DOI:** 10.1017/cts.2023.659

**Published:** 2023-11-03

**Authors:** Sarah C. Lotspeich, Bryan E. Shepherd, Marion Achieng Kariuki, Kara Wools-Kaloustian, Catherine C. McGowan, Beverly Musick, Aggrey Semeere, Brenda E. Crabtree Ramírez, Denna M. Mkwashapi, Carina Cesar, Matthew Ssemakadde, Daisy Maria Machado, Antony Ngeresa, Flávia Faleiro Ferreira, Jerome Lwali, Adias Marcelin, Sandra Wagner Cardoso, Marco Tulio Luque, Larissa Otero, Claudia P. Cortés, Stephany N. Duda

**Affiliations:** 1 Department of Statistical Sciences, Wake Forest University, Winston-Salem, NC, USA; 2 Department of Biostatistics, Vanderbilt University Medical Center, Nashville, TN, USA; 3 Infectious Diseases Institute, Makerere University, Kampala, Uganda; 4 Department of Medicine, Indiana University School of Medicine, Indianapolis, IN, USA; 5 Division of Infectious Diseases, Department of Medicine, Vanderbilt University Medical Center, Nashville, TN, USA; 6 Department of Biostatistics, Indiana University School of Medicine, Indianapolis, IN, USA; 7 Department of Infectious Diseases, Instituto Nacional de Ciencias Méxicas y Nutrición Salvador Zubirán, Mexico City, Mexico; 8 Sexual and Reproductive Health Program, National Institute for Medical Research Mwanza, United Republic of Tanzania, Mwanza, Tanzania; 9 Fundación Huésped, Buenos Aires, Argentina; 10 Masaka Regional Hospital, Masaka, Uganda; 11 Departamento de Pediatria, Universidade Federal de São Paulo, São Paulo, Brazil; 12 Academic Model Providing Access to Health Care (AMPATH), Eldoret, Kenya; 13 Departamento de Pediatria, Universidade Federal de Minas Gerais, Belo Horizonte, Brazil; 14 Tumbi Hospital HIV Care and Treatment Clinic, United Republic of Tanzania, Kibaha, Tanzania; 15 Le Groupe Haïtien d’Etude du Sarcome de Kaposi et des Infections Opportunistes, Port-au-Prince, Haiti; 16 Instituto Nacional de Infectologia Evandro Chagas, Rio de Janeiro, Brazil; 17 Instituto Hondureño de Seguridad Social and Hospital Escuela Universitario, Tegucigalpa, Honduras; 18 Instituto de Medicina Tropical Alexander von Humboldt, Universidad Peruana Cayetano Heredia, Lima, Peru; 19 School of Medicine, Universidad Peruana Cayetano Heredia, Lima, Peru; 20 Unidad Médica, Fundación Arriarán, Santiago, Chile; 21 Department of Biomedical Informatics, Vanderbilt University Medical Center, Nashville, TN, USA

**Keywords:** Data audits, data quality, HIV, international research, LMIC, observational cohorts

## Abstract

**Introduction:**

Routine patient care data are increasingly used for biomedical research, but such “secondary use” data have known limitations, including their quality. When leveraging routine care data for observational research, developing audit protocols that can maximize informational return and minimize costs is paramount.

**Methods:**

For more than a decade, the Latin America and East Africa regions of the International epidemiology Databases to Evaluate AIDS (IeDEA) consortium have been auditing the observational data drawn from participating human immunodeficiency virus clinics. Since our earliest audits, where external auditors used paper forms to record audit findings from paper medical records, we have streamlined our protocols to obtain more efficient and informative audits that keep up with advancing technology while reducing travel obligations and associated costs.

**Results:**

We present five key lessons learned from conducting data audits of secondary-use data from resource-limited settings for more than 10 years and share eight recommendations for other consortia looking to implement data quality initiatives.

**Conclusion:**

After completing multiple audit cycles in both the Latin America and East Africa regions of the IeDEA consortium, we have established a rich reference for data quality in our cohorts, as well as large, audited analytical datasets that can be used to answer important clinical questions with confidence. By sharing our audit processes and how they have been adapted over time, we hope that others can develop protocols informed by our lessons learned from more than a decade of experience in these large, diverse cohorts.

## Introduction

Routine patient care data are used increasingly for biomedical research because they provide a variety of clinical and demographic variables on a large number of diverse patients seeking treatment for specific conditions or within specific geographic locations. Furthermore, “secondary use” of such data incurs minimal data collection costs for research. In human immunodeficiency virus (HIV) care, clinic-based cohorts generate large amounts of routine clinical and laboratory data that may be pooled to assess global trends in HIV care and treatment. However, since they were collected primarily for clinical care and billing purposes, such secondary-use data have known limitations. Limitations include data collection biases and errors in completeness and accuracy, as these data are usually not subject to the same systematic practices used in research data collection [1,2]. These errors may affect the validity of studies that rely on such data to inform clinical practice and public policy or to help design study interventions [3].

Research teams and clinical trial monitors often conduct quality assurance monitoring and data audits to understand the reliability of research data sources. In the scope of this paper, quality assurance monitoring and data auditing for observational studies differ primarily in their timing and impact on analyses. Quality assurance monitoring is an ongoing process and does not involve comparing data to source documents. Instead, data managers send data queries about missing, outlying, and invalid data points and receive responses and corrected datasets. Knowledge of these corrections is not incorporated into statistical analysis; the final versions of the datasets are used “as is.” Audits occur much more infrequently, involve comparing data to source documents, and result in calculated error rates that can be incorporated into statistical analyses. This paper discusses the use of auditing for data quality in observational studies.

The typical audit process involves trained auditors from an external, independent review team visiting a research site, reviewing original clinical source documents (e.g., paper charts, electronic lab systems) for a subset of patients, and recording any discrepancies between the research database and the source documents. Although in-person data auditing has long been standard practice [4], the increasing availability of internet and electronic health record (EHR) systems has allowed auditors in some settings to conduct remote auditing, whereby they receive a limited-access login to the EHR to review electronic source data without requiring a site visit [5,6]. The use of remote monitoring for clinical trials is increasing in high-resource settings, particularly given the COVID-19 pandemic [7,8].

Audit methods designed for clinical trials have been adapted for observational studies [e.g., 3,9–17], and can help elucidate the accuracy and completeness of the clinical data being repurposed for research. Audits in observational studies can also identify errors in data extraction routines from clinical systems, highlight areas for improvement in data management practices or data collection methods, and act as deterrents against fraud. Still, one-hundred-percent source document verification for large datasets is both time- and cost-prohibitive [18]. Fortunately, the following can provide improved approaches to data quality assessment: (i) risk-based auditing [19–20], which allows for customizing audits to prioritize monitoring of high-risk activities, study processes, site components, or data points; (ii) new audit designs that strategically target the most informative records for validation [e.g., 21–25]; and (iii) statistical methods for addressing errors that incorporate both audit and original data into analyses, which can recover unbiased and statistically efficient estimates [e.g., 26–29].

Despite clear benefits, there remain challenges to implementing audit methods for secondary-use datasets, particularly for multi-site studies conducted in resource-limited settings. Audits in such settings may be more likely to require on-site rather than remote monitoring, given weak or inconsistent internet availability, a lack of secure EHR remote access protocols or capacity, or paper-based systems. Auditors require funding for travel and personnel time, and may not be familiar with local medical records, data systems, or procedures [30]. Additional challenges to auditors include language barriers and interpreting handwritten clinical notes. These external audits can also be demanding of on-site investigators, who need to prepare for the audit by organizing medical records; obtaining visitor access to paper charts, electronic systems, and the hospital internet system (if applicable); and hosting an audit team. These concerns are magnified in multi-national research networks, where language and cultural differences add complexity.

The Latin America and East Africa regions of the International epidemiology Databases to Evaluate AIDS (IeDEA) consortium have been conducting audits of observational data drawn from participating HIV clinics for more than a decade. Since our earliest on-site audits in 2007, where we used paper forms to record audit findings from paper medical records, we have streamlined the process to allow for more efficient and informative audits to keep up with advancing technology while reducing travel obligations. In this manuscript, we describe the evolving audit processes implemented in two multi-national HIV cohorts (Section 2), present lessons learned from conducting data audits of secondary-use data from resource-limited settings for more than 10 years (Section 3), and share recommendations for other consortia looking to implement data quality initiatives (Section 4).

## Materials and Methods

### Cohort Profiles

The IeDEA consortium brings together 388 HIV clinics across seven geographic regions for the large-scale aggregation of high-quality observational clinical HIV data, which can be used to answer key questions that may be unanswerable with only a single cohort [31]. Each member region of IeDEA includes multiple clinical sites and a regional data center (RDC) that is responsible for merging the data, preparing analytical datasets, and monitoring data quality across its sites.

Two of the IeDEA regions have formalized data quality programs with over 10 years of data audit cycles: East Africa IeDEA (EA-IeDEA) [32] and the Caribbean, Central, and South America network for HIV epidemiology (CCASAnet) [33]. Both regions collect routine care data on children, adolescents, and adults living with HIV, including demographic, laboratory, medication, and other clinical data. These data are initially collected by doctors, nurses, and other healthcare staff, and they are recorded in either electronic medical records or paper charts. Data are subsequently digitized/entered into the research database for annual submission to the RDC. Over time, some sites have added dedicated staff for data management while others have reduced the numbers of data personnel given increasing use of computer-based systems.

The CCASAnet RDC is located at Vanderbilt University Medical Center in Nashville, Tennessee, USA, while the EA-IeDEA RDC is based at Indiana University, Indianapolis, USA, with a partner data center in Kenya. As of 2022, the pooled CCASAnet database encompassed data from approximately 55,000 persons living with HIV from sites in Argentina, Brazil, Chile, Haiti, Honduras, Mexico, and Peru, and EA-IeDEA included data on over 505,000 persons living with HIV from Kenya, Tanzania, and Uganda. Although a small number of sites have left the networks over time (e.g., due to funding challenges, changes in leadership, reduced capacity for research, and inadequate data gathering), the variables gathered and harmonized have remained consistent. To support data harmonization across regions, database structures for both regional cohorts are modeled after the HIV Cohorts Data Exchange Protocol and the IeDEA Data Exchange Standard [34,35]. Clinical source documents at participating HIV clinics include both paper-based patient charts and EHR systems. Data audits are part of the CCASAnet and EA-IeDEA protocols approved by the Institutional Review Boards at Vanderbilt University Medical Center and Indiana University, respectively. Protocols are also approved by the respective ethics committees of the CCASAnet and EA-IeDEA member institutions.

### Audit Process

#### Audit history

To date, both CCASAnet and EA-IeDEA have each completed three cycles of audits across sites participating in their networks. The CCASAnet RDC at Vanderbilt began routinely conducting on-site audits in July 2007, at which time there were eleven participating clinical sites located in Argentina, Brazil, Chile, Haiti, Honduras, Mexico, and Peru. The EA-IeDEA RDC conducted its first audits in April 2010, at which time there were twelve participating clinical programs located in Kenya, Tanzania, and Uganda, with multiple sites within each program. Since then, both RDCs have conducted audits approximately every 2–3 years to continually assess data quality in their expanding networks. Specifically, the CCASAnet audit cycles took place in 2007–2010, 2012–2014, and 2016–2018; the EA-IeDEA audit cycles were conducted in 2010–2011, 2012–2014, and 2017–2019. Information about individual audit cycles, including audit dates, sample sizes, and numbers of audited variables, is summarized in Tables S1 and S2 (Supplementary Material 1) for CCASAnet and EA-IeDEA respectively. Audit sites have been anonymized here and are represented as Sites C1-C10 in CCASAnet and Sites E1–E14 in EA-IeDEA.

#### General audit procedures

The on-site audit procedure implemented in the first CCASAnet audit cycle is described in detail elsewhere [13]. Briefly, each RDC selected a random sample of approximately 20–30 patient records from each participating site to be reviewed. The number of patient records selected was limited by the time constraints/duration of the site visit. Sites were notified in advance about most of these selected records, so that they could locate source documents prior to the auditors’ arrival. Upon arrival, the auditors provided a list of five to ten additional records for auditing; this was included as a validity check to ensure that records were not altered in advance. A team of investigators from the RDC made up of at least one clinician and one informatician traveled to each site for a period of 2–3 days. During the audit, the auditors compared the selected records’ source documentation (e.g., patient charts, electronic health records, laboratory, and pharmacy databases) to the same entries in the research dataset submitted to the RDC. On the final day of the audit, the audit team met with site personnel, including the site Principal Investigator, to present preliminary findings, discuss the site’s overall data quality, provide guidance on quality improvement procedures, and solicit feedback regarding the audit process. The RDC subsequently prepared a written report and provided it to the site. The EA-IeDEA RDC made small modifications to the procedure, conducting audits at the program level as some of the program’s individual sites were small, difficult-to-access rural facilities with few patients, and involving local clinicians and data managers in the audit process to provide local context. Both RDCs maintained standardized procedures and training materials for data review that guided the conduct of audits.

#### Variables audited

The baseline audits in both CCASAnet and EA-IeDEA focused on core clinical variables, including basic patient information (e.g., sex, dates of birth and death), visit information (e.g., visit date, patient weight), lab values (e.g., CD4 lymphocyte counts [CD4], hemoglobin, HIV viral load values, lab-associated dates), HIV disease stage classification, and antiretroviral therapy information (e.g., individual antiretrovirals used and associated start and end dates). In successive audit cycles, as the collection of HIV care and treatment data expanded, the number of unique clinical variables in both programs’ audits expanded to include other data, such as non-communicable diseases, tuberculosis (TB) diagnosis and treatment, AIDS diagnosis, receipt of antiretroviral therapy prior to clinic enrollment, pregnancy, and family planning variables.

#### Selection of records for auditing

Processes for audit record selection changed over time in both audit programs. Initially, all audit records were selected randomly within sites/programs. However, the baseline CCASAnet audits (Cycle 1) revealed between-site variability in data quality (Table S2 in Supplementary Material 1). These results informed the selection of clinical sites for the Cycle 1 “re-audits” of sites with identified data quality challenges, as well as the number of records sampled in subsequent audit cycles. Given resource constraints, these re-audits were allocated to sites with higher error rates (>15%) in key variables and unclear causes, solutions, or explanations for the errors. Cycle 2 audits for both CCASAnet and EA-IeDEA targeted newer data by focusing on patients with at least one clinic visit in the past year. This shifted the audits towards more current patient records, which better reflected ongoing programmatic changes in HIV care and treatment and enabled quality assessment of recent data that were more pertinent to planned analyses. In Cycle 3 of CCASAnet, a random sample of Cycle 2 records was included to ensure that previously detected errors had been addressed. Also in Cycle 3, both RDCs oversampled adults and children diagnosed with Kaposi’s sarcoma (KS) and/or TB, corresponding to specific research studies.

#### Audit tools

From the beginning, the RDCs for CCASAnet and EA-IeDEA have used different tools to conduct their audits. During CCASAnet Cycle 1, auditors recorded their findings for each variable on standardized paper audit forms, using one form per patient. Audit results then had to be manually transcribed to construct the analytical dataset. In CCASAnet Cycle 2, a custom-built computer application was developed and introduced at some sites to record and tabulate audit findings and reduce the burden of manual transcription of findings. Using the Research Electronic Data Capture (REDCap) software [36], the RDC created a reusable auditing tool adapted from previous audit templates [13,37]. Data from selected patient records were extracted for the audit from the most recent version of the CCASAnet research database, then imported and formatted for review in the REDCap database. This extraction took place prior to the start of the audits. Record review took place directly therein: auditors compared the extracted value in REDCap (the original) to source documentation in the patient chart and categorized their findings into one of the audit codes (e.g., “matches chart,” “doesn't match chart,” “can't find in chart,” or “new entry found that was missing from research database”). Upon indicating that a variable was erroneous (including those that did not match the chart or new data found that were not in the research database), auditors were prompted to submit the corrected value in a format-validated field (e.g., date, integer, dropdown menu) aligned with the variable type. Auditors would access the REDCap database at sites where reliable internet was available; otherwise, sites were audited using paper forms. Following completion of the audit, investigators at Vanderbilt extracted the data directly from REDCap into R for analysis, error rate calculations, and reporting. With minor improvements, the same electronic audit tool was used for all sites in CCASAnet Cycle 3. A copy of the REDCap project data dictionary (CSV format) used for the 2017 CCASAnet audits (Cycle 3) is included in Supplementary Material 2. The visit, medication, laboratory, and clinical endpoint forms should be enabled as “repeating instruments” in REDCap.

EA-IeDEA used Excel spreadsheets that were pre-populated with the data from the research database for all cycles. Auditors entered all found values from the source documentation in a parallel column during the audit process, yielding two versions of each variable (original and verified). These two columns were subsequently compared, and discrepancies were categorized as “mismatched” or “unverifiable,” which correlate to findings of “doesn’t match chart” or “can’t find in chart,” respectively, in CCASAnet. When a discrepancy was identified, the auditor would insert a note in the patient’s chart indicating that an entry error was detected or that a data point had not yet been entered. Following completion of the audit, investigators at the EA-IeDEA RDC imported the data from Excel into SAS for analysis, error rate calculations, and reporting.

#### Self-audits

Most recently, the CCASAnet and EA-IeDEA audit protocols have shifted away from traditional on-site monitoring such that sites now designate their own investigators to be trained and carry out the audit themselves (called “self-audits”). Local teams in both CCASAnet and EA-IeDEA had prior experience with conducting audits. During CCASAnet Cycle 1, site teams were trained to independently replicate Cycle 1 audits to ensure the quality of the Vanderbilt RDC audit. Beginning in the early audit cycles in East Africa, the audit teams at some sites included site-level data managers and investigators. Site-level data managers were involved in all cycles of audits in East Africa, although their roles evolved over time.

This self-audit design allowed for the collection of large audit datasets from more clinical sites using fewer resources than were required by previous on-site external audits (“travel-audits”). The comparative efficacy of the self- and travel-audits in CCASAnet was discussed in detail previously [16], where we concluded that the proposed self-audits were an effective alternative to conventional on-site monitoring by the RDC (travel-audits) for audit-experienced sites. Anecdotally, the EA-IeDEA experience has been similar.

## Results

### Audit Findings

CCASAnet Cycle 1 (April 2007–March 2010) included baseline on-site audits at ten clinics followed by subsequent re-audits at four of them, i.e., a follow-up audit for a subset of sites found to have more error-prone data. Most variables included in the research database agreed with original patient charts (*n* = 4241; 82%), while approximately 7% of variables did not match the patient chart, 7% could not be located, and 3% were missing from the research database. EA-IeDEA Cycle 1 audits were conducted on-site at twelve clinics between 2010 and January 2011. More variables included in the EA-IeDEA research database agreed with patient charts (*n* = 51,422; 95%), possibly because the audit included a different mix of variables and the source documents were more structured, with less free-text subject to interpretation. In the EA-IeDEA Audit, only 4% of variables did not match the patient chart and 2% could not be located in the chart.

Both CCASAnet and EA-IeDEA had between-site variability in audit findings in Cycle 1. In CCASAnet, the percentages of values at a site correctly matching source documents ranged from 61% to 96%. Later in CCASAnet’s Cycle 1, the sites selected for re-audit had (absolute) improvements of 8%–9% in agreement with patient charts. In EA-IeDEA, the site-specific percentages of values correctly matching source documents varied between 75% and 98% across clinics.

By the Cycle 2 audits, both networks had lost and gained participating HIV care and treatment centers, slightly altering the list of audited sites. CCASAnet Cycle 2 audits (December 2012–April 2014) revealed a 5% increase in the overall, all-sites rate of variables matching patient charts (up to 87% from 82% in Cycle 1) and reduced variability in the site-specific error rates as they ranged from 82% to 94% correct data (a between-site difference of at most 12% versus 35% in Cycle 1), suggesting improvement across the network. Auditors in EA-IeDEA Cycle 2 (May 2011–February 2014) reported a slight decrease in the all-site rate of matching variables (down to 92% from 95% in Cycle 1), likely owing to the addition of many previously unaudited variables to the audit protocol. As with CCASAnet, there was less variability in site-specific error rates, with between 78% and 96% correct data at each site (a between-site difference of at most 18% versus 23% in Cycle 1).

**Figure 1.**
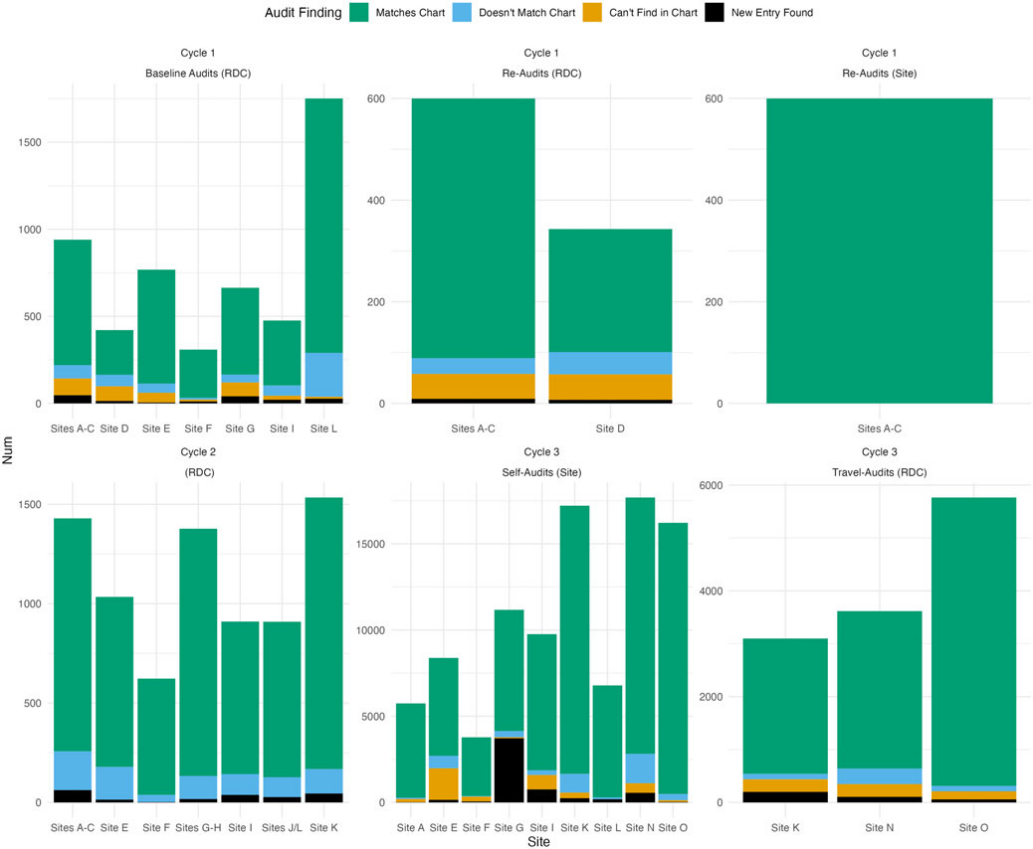
Breakdown of overall data quality according to separate CCASAnet audit projects over time. (RDC or site in parentheses indicates that the audits were conducted by the regional data coordinating center at Vanderbilt University or site investigators, respectively).

**Figure 2.**
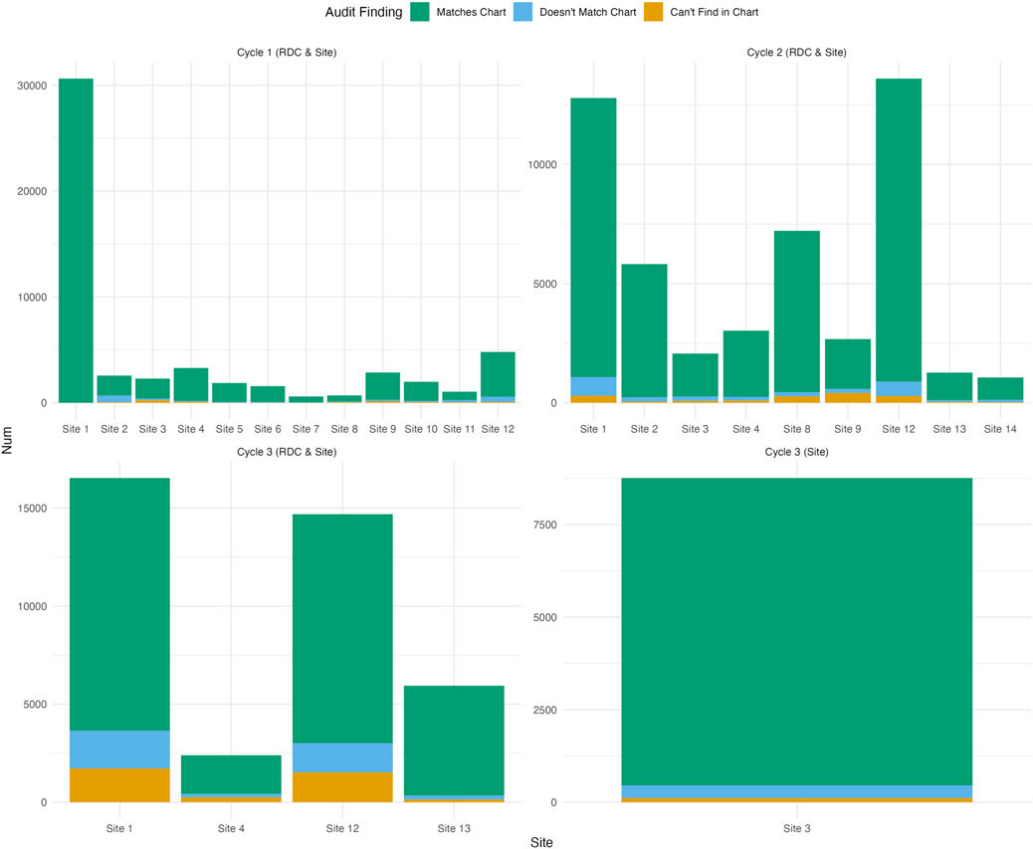
Breakdown of overall data quality according to separate EA-IeDEA audit projects over time. (RDC or site in parentheses indicates that the audits were conducted by the regional data coordinating center at Indiana University or site investigators, respectively).

The Cycle 3 audits for CCASAnet and EA-IeDEA found similar error patterns as Cycle 2, despite adding new categories of previously unaudited data. Cycle 3 audit methods were less comparable between the RDCs, given CCASAnet’s switch to self-audits and EA-IeDEA’s focus on a subset of clinics. With CCASAnet’s exclusive adoption of the REDCap audit tools, Cycle 3 also saw a noticeable increase in the total number of audited variables, from 7349 across 11 sites to 96,837 (self-audited) across 9 sites. Overall data quality findings from CCASAnet and EA-IeDEA audits are presented in Fig. 1 and Fig. 2, respectively. Additional details are reported in Supplementary Material 1.

### Lessons Learned

For more than a decade, we have been honing our audit protocols in CCASAnet and EA-IeDEA to maximize the information gained from partial data audits on a fixed research budget. In the current work, we describe how our protocols have evolved alongside the changing biomedical landscape of our clinical sites and benefited from the availability of new technology to support web-based audit reporting. We share our lessons learned so that other cohorts might benefit from our experiences when designing their own data quality initiatives.

#### Audits are critical to understanding data and processes

The audit process is vital for data coordinating centers to understand the nuances of data collection, recording, and abstraction at their sites, and it can be adapted from the clinical trials framework for observational cohort studies like CCASAnet and EA-IeDEA. Only when actually comparing parts of the research database to the original source documents, do we fully grasp the process involved in transforming these data to align with a fixed set of standards (like the HIV Cohorts Data Exchange Protocol or the IeDEA Data Exchange Standard). Indeed, observational data are often collected with other objectives (e.g., immediate patient care or billing) in mind. Therefore, audits are critical for identifying systematic errors, which are often due to ineffective data collection workflows; lack of abstractor training; errors in clinical software or data processing scripts; miscoding, rounding, or overlooking certain forms in the patient chart/EHR; relying only on a subset of source documents, such as HIV clinic charts and not hospital records, for complete data; and misunderstanding clinical content. After such errors are uncovered, they can be addressed by the sites and/or the RDC to improve future data collection or abstraction procedures.

As one example, during the EA-IeDEA Cycle 1 audits, auditors at one clinic discovered that CD4 values were not being recorded on the date that the blood was drawn but rather on the date that the patient returned to the clinic to receive results or attend a subsequent visit. This inconsistency created bias in the dataset; CD4 values on the sickest and most immune-suppressed patients were sometimes not being recorded as they often did not return to the clinic due to illness or death. The audit findings prompted all HIV clinics in the country to undertake a massive review of their CD4 data, leading to revised standardized data collection forms and personnel training around the entry of lab results. Without the audit, this issue would have been discovered and corrected much later, if ever. CCASAnet audits similarly discovered errors in the programing of one hospital’s electronic pharmacy system, where entry of new prescriptions was partially overwriting a patient’s historical medication data. The audit findings led to fixes in the software, preserving future records from data loss.

Audits are particularly beneficial and informative when onboarding new sites, initiating studies that rely on new types of data, or following major process changes, such as the introduction of a new EHR. Although there may be diminishing returns of repeated audits when data quality seems stable, there are still benefits to continued auditing. For example, one CCASAnet site had a decrease in data quality after previously having high data quality; this was a surprising finding, identified to be the result of EHR export glitches and inexperienced student data abstractors. Still, we have observed improvements in the stability of data workflows and the quality of re-audited data, as seen across Tables S1 and S2 (Supplementary Material 1).

#### Paper is bad; electronic audit systems are better

The first rounds of CCASAnet audits were on paper. Auditors from the CCASAnet RDC carried printed forms to all of their on-site visits, leaving with stacks of completed audit forms that were handwritten and included many abbreviations. Because of this, generating rapid post-audit reports was a laborious process: the review of paper audit forms and manual tabulation of errors for hundreds of variables was time-consuming and error-prone in itself. EA-IeDEA auditors, who have used Excel throughout, avoided many of these paper-related problems, but also contended with post-audit interpretation of free-text fields in Excel when trying to generate summary reports. More recently, EA-IeDEA wrote SAS code that has made the reporting process easier and more efficient. CCASAnet audits in Cycle 2 and beyond have used REDCap-based forms, which has alleviated many of these problems. There is no longer a need to carry paper forms, auditors are forced to enter audit data in a structured form, and summary reports can be more easily generated. However, REDCap audit data entry has not been without its hiccups. For example, the first REDCap audit form required so much clicking that one of the auditors said it was “as bad as our outdated EHR.” Improvements have since been made to further streamline the REDCap audit forms. Overall, electronic audit capture tools have streamlined the following processes: (i) comparison of extracted pre-audit data to source documentation, (ii) submission of findings or corrections according to formal error categories, and (iii) export of the audit data for analysis and quality reporting.

#### Auditors are not always correct

Audits are best performed with a healthy level of humility, recognizing that auditors make mistakes and that the interpretation of source documents in varied, international settings may require additional nuance. Furthermore, the audit data are not always correct. For example, in CCASAnet Cycle 1, the RDC encountered problems when pre-populating the paper audit forms with the data from the sites, so, occasionally, auditors found errors in the forms that were the fault of the RDC, not the site. Similarly, when testing procedures for the self-audit, findings from the external travel-auditors and the internal self-auditors occasionally differed, either due to reasonable differences in data interpretation, an imprecise variable definition, or a mis-recording of data by travel-auditors. EA-IeDEA’s approach of involving a local investigator in all audits may help to minimize data misinterpretation and foster trust.

#### Self-audits should be considered

On-site auditing (travel-audits) is expensive and time-consuming. Many more records can be audited by local site investigators, and, if there is trust between them and the RDC, this can be a collaborative way to promote data quality. The CCASAnet self-audit model worked well, was low cost, and turned out to be particularly useful during restricted travel beginning in 2020 related to the COVID-19 pandemic. Involving local investigators to work alongside RDC investigators during travel-audits can provide excellent training for the future conduct of self-audits. At one EA-IeDEA site, the site data manager, who had directly participated in previous travel-audits, went on to complete the self-audit on his own. However, we acknowledge that self-audits are likely inadequate for new study sites or new types of data collection because the RDC must first understand workflows at a site, establish baseline data quality, and promote a culture of data quality assessment. For best results, these self-audits should be done in addition to ongoing site-based data quality assurance monitoring.

#### Audits need to adapt over time

Over time, the audit processes of the RDCs have evolved to learn as much about our regions as we can efficiently and with maximal cost-effectiveness by (i) targeting the most informative records to audit and (ii) streamlining our audit protocol. For example, CCASAnet and EA-IeDEA are longitudinal studies, but reviewing every visit in patient records with long follow-up periods is cost-prohibitive. Instead, audits can focus on impactful time periods, such as those capturing new diagnoses or modifications in clinical care. Similarly, once a certain variable is found to be of fairly good quality, there are limited benefits in repeatedly reviewing it. Lab values are a good example of this, as auditing all lab values can be tedious (some patients have as many as 150 CD4+ test results), and these data are usually accurate because, even at sites with paper charts, lab data are often sourced directly from electronic systems. Other data points like antiretroviral therapy regimens, other medications, and disease diagnoses are more error-prone, and auditing these variables is typically a better use of auditors’ time.

## Conclusion

### Recommendations

Based on these lessons learned, we offer eight core recommendations for other research networks.

**R1:** Observational research cohorts should implement data quality audits, too, not just clinical trials, and audit protocols must be adapted for feasibility in resource-limited settings.

**R2:** Data coordinating centers should prioritize the first (i.e., “baseline”) audit, as it provides data and workflow insights and can lead to transformations with paper charting or EHR use, processes, and staff. In addition, an in-person visit from RDC investigators can help establish connections with local investigators and staff.

**R3:** Subsequent major changes in clinic systems, personnel, or collected study data can introduce errors and should trigger re-audits.

**R4:** Site personnel should be trained to understand the data quality assurance process and conduct their own (self-)audits. Creation of site-based data quality programs can build trust, improve attentiveness to data detail and quality, positively impact local data operations, and build capacity for future site-based research.

**R5:** After establishing trust and stable processes, the coordinating center can implement lower-cost solutions, such as self-audits and remote auditing.

**R6:** The content of the audits should change over time to ensure high data quality for the ongoing cohort research. These changes may include auditing different variables or subsets of patients. Also, repeatedly auditing the same content can have diminishing returns.

**R7:** Paper audit forms should be avoided. Electronic systems like REDCap or Excel produce more reusable audit findings that can be used to generate comparisons and summary reports in a timely manner and avoid potential transcription error.

**R8:** Coordinating centers for multi-national collaborative research consortia should realize that there is no perfect data audit and there are many potential interpretations of audit findings. The goal is to learn lessons about data in different contexts and improve quality through collaboration.

### Strengths and Limitations

Strengths of this study include the extensive time period, geographic scope, and overall sample size of our data audits. However, we recognize that every research cohort is different, and our recommendations, designed for research cohorts where study data are manually extracted from clinical source documents, may not apply to more highly resourced-research settings. Indeed, almost all clinics in these networks still maintain paper clinical charts, so the protocols outlined here might not directly apply to a setting exclusively using EHRs or other paperless data capture. In addition, some of our audit protocols, especially the self-audits, are not intended as first steps for a cohort that is new to data collection and data quality concepts. Further, these internal reviews are not advised when there is concern about scientific misconduct or fraud.

As the importance of data quality comes to the forefront of observational research, developing audit protocols that can maximize informational return and minimize costs is essential. With multiple cycles of audits completed in both CCASAnet and EA-IeDEA since 2007, we have established a rich reference for data quality in our cohorts and curated large, audited analytical datasets that can be used to answer important clinical questions with confidence. By sharing our audit processes and how they have adapted over time, we hope that others can develop protocols informed by our lessons learned from more than a decade of experience in these large, diverse cohorts.

## Supporting information

Lotspeich et al. supplementary material 1 Lotspeich et al. supplementary material

Lotspeich et al. supplementary material 2 Lotspeich et al. supplementary material

## Supplementary material

The supplementary material for this article can be found at https://doi.org/10.1017/cts.2023.659.
