# Supplementary material for: Lessons learned from over a decade of data audits in international observational HIV cohorts in Latin America and East Africa
Source: J Clin Transl Sci. 2023 Nov 3;7(1):e245. doi: 10.1017/cts.2023.659 (PMC10685260; doi:10.1017/cts.2023.659)
Supplement: Lotspeich et al. supplementary material 1 — Lotspeich et al. supplementary material [file S2059866123006593sup001.docx]

**Supplemental Materials for** “​​​​**Lessons learned from over a decade of data audits in international observational HIV cohorts in Latin America and East Africa”**

# Sarah C. Lotspeich, Bryan E. Shepherd, Marion Achieng Kariuki, Kara Wools Kaloustian, Catherine C. McGowan, Beverly Musick, Aggrey Semeere, Brenda E. Crabtree Ramírez, Denna M. Mkwashapi, Carina Cesar, Matthew Ssemakadde, Daisy Maria Machado, Antony Ngeresa, Flávia G. Faleiro Ferreira, Jerome Lwali, Adias Marcelin, Sandra Wagner Cardoso, Marco Tulio Luque, Larissa Otero, Claudia P. Cortés, Stephany N. Duda

**Table S1.** Detailed breakdown of the CCASAnet audit projects (by site) and the data collected therein.

|  | **Auditors** | **Date** | **Audited records** | **Clinical variables reviewed** | **Audited variables** |
| --- | --- | --- | --- | --- | --- |
| **Cycle 1** |  | **Sep 2007–Mar 2010** | **276** | **129** | **6689** |
| *Baseline Audits* | *RDC* | *Sep 2007–Apr 2008* | *193* | *75* | *5144* |
| Sites A–C |  | Sep 2007 | 31 | 11 | 941 |
| Site D |  | Nov 2007 | 18 | 11 | 421 |
| Site E |  | Jul–Aug 2007 | 26 | 11 | 768 |
| Site F |  | Apr 2007 | 17 | 9 | 325 |
| Site G |  | Apr 2008 | 33 | 11 | 664 |
| Site I |  | Apr 2008 | 29 | 11 | 501 |
| Site L |  | Jul 2007 | 39 | 11 | 1524 |
| *Re-Audits* |  | *May 2008, Mar 2010* | *83* | *54* | *1545* |
| Sites A–C | Site | Mar 2010 | 25 | 20 | 600 |
|  | RDC | Mar 2010 | 25 | 20 | 600 |
| Site D |  | May 2008 | 33 | 14 | 345 |
| **Cycle 2** | **RDC** | **Dec 2012–Aug 2014** | **250** | **252** | **7349** |
| Sites A–C |  | Mar 2013,  May–Jun 2014 | 43 | 37 | 1429 |
| Site E |  | Sep 2013; Aug 2014 | 34 | 34 | 1034 |
| Site F |  | Apr 2013 | 28 | 26 | 623 |
| Sites G–H |  | Jul 2013 | 60 | 34 | 910 |
| Site I |  | Dec 2012 | 24 | 34 | 910 |
| Sites J/L |  | Feb 2013, Jul 2014 | 30 | 40 | 909 |
| Site K |  | Jul 2013, Apr 2014 | 31 | 47 | 1534 |
| **Cycle 3** |  | **Jun 2016–Jun 2018** | **479** | **450** | **109276** |
| *Self-Audits* | *Site* | *Jun 2016–Jun 2018* | *414* | *333* | *96837* |
| Site A |  | May–Jun 2018 | 28 | 28 | 5744 |
| Site E |  | Jun–Jul 2016 | 50 | 39 | 8381 |
| Site F |  | Jun–Jul 2016 | 56 | 33 | 3779 |
| Site G |  | Jun–Jul 2016 | 50 | 41 | 11171 |
| Site I |  | Jun–Jul 2016 | 50 | 41 | 9761 |
| Site K |  | Jun–Jul 2016 | 50 | 39 | 17211 |
| Site L |  | Jun–Jul 2016 | 50 | 34 | 6787 |
| Site N |  | Jun–Jul 2016 | 40 | 41 | 17686 |
| Site O |  | Jun–Jul 2016 | 40 | 37 | 16317 |
| *Travel-Audits* | *RDC* | *Jun–Jul 2017* | *65* | *117* | *12439* |
| Site K |  |  | 25 | 40 | 5765 |
| Site N |  |  | 19 | 40 | 3617 |
| Site O |  |  | 21 | 37 | 3057 |

**Site** represents the HIV care and treatment clinic that was audited. **Auditors** refer to either a team of auditors from the CCASAnet Data Coordinating Center at Vanderbilt (composed of at least one clinician and one informatician) and/or data management delegates from the clinical site. **Audited records** captures the number of patients’ records that were at least partially reviewed. **Clinical variables reviewed** is the number of unique clinical variables reviewed (e.g. date of birth, HIV diagnosis date, HIV viral load test result). **Audited variables** denotes the total number of data points audited (across all patients, all variables).

**Table S2.** Detailed breakdown of the East Africa IeDEA audit projects (by site) and the data collected therein.

|  | **Auditors** | **Date** | **Audited records** | **Clinical variables reviewed** | **Audited variables** |
| --- | --- | --- | --- | --- | --- |
| **Cycle 1** | **RDC & Site** | **Apr 2010–Jan 2011** | **265** | **386** | **54209** |
| Site 1 |  | Aug 2010 | 43 | 88 | 31367 |
| Site 2 |  | Aug 2010 | 20 | 37 | 2045 |
| Site 3 |  | May 2010 | 22 | 36 | 2077 |
| Site 4 |  | Jun 2010 | 12 | 21 | 3287 |
| Site 5 |  | Aug 2010 | 19 | 29 | 1867 |
| Site 6 |  | May 2010 | 12 | 28 | 1574 |
| Site 7 |  | May 2010 | 8 | 27 | 597 |
| Site 8 |  | Apr 2010 | 12 | 21 | 695 |
| Site 9 |  | Apr 2010 | 22 | 24 | 2865 |
| Site 10 |  | Apr 2010 | 24 | 25 | 1986 |
| Site 11 |  | May 2010 | 25 | 20 | 1047 |
| Site 12 |  | Jan 2011 | 46 | 30 | 4802 |
| **Cycle 2** | **RDC & Site** | **May 2011–Feb 2014** | **252** | **479** | **49514** |
| Site 1 |  | Nov 2012 | 58 | 90 | 12787 |
| Site 2 |  | Aug 2012 | 19 | 62 | 5819 |
| Site 3 |  | Feb 2012 | 19 | 58 | 2066 |
| Site 4 |  | Feb 2012 | 20 | 46 | 3026 |
| Site 8 |  | May 2011 | 18 | 43 | 7215 |
| Site 9 |  | May 2011 | 19 | 43 | 2672 |
| Site 12 |  | Aug 2012 | 73 | 62 | 13601 |
| Site 13 |  | Feb 2012 | 11 | 47 | 1266 |
| Site 14 |  | Feb 2014 | 15 | 28 | 1062 |
| **Cycle 3** |  | **Nov 2017–Mar 2019** | **138** | **378** | **48308** |
| Site 1 | RDC & Site | Oct 2018–Mar 2019 | 57 | 98 | 16530 |
| Site 4 | RDC & Site | Nov 2017 | 15 | 53 | 2393 |
| Site 12 | RDC & Site | Nov 2018 | 30 | 72 | 14686 |
| Site 13 | RDC & Site | Nov 2017 | 13 | 65 | 5941 |
| Site 3 | Site | Nov 2017 | 23 | 90 | 8758 |

**Site** represents the HIV clinical site that was audited. **Auditors** refer to a team of auditors from the EA-IeDEA Data Coordinating Center at Indiana University and data management delegates from the clinical site. **Audited records** captures the number of patients’ records that were at least partially reviewed. **Clinical variables reviewed** is the number of unique clinical variables reviewed (e.g., date of birth, HIV diagnosis date, HIV viral load test result). **Audited variables** denotes the total number of data points audited (across all patients, all variables).
